# Facilitating regular Physical Education for students with disability—PE teachers' views

**DOI:** 10.3389/fspor.2024.1400192

**Published:** 2024-07-31

**Authors:** Karin Bertills, Maria Björk

**Affiliations:** ^1^ Department of Behavioural Sciences and Learning, Division of Psychology, Linköping University, Linköping, Sweden; ^2^ Department of Nursing, School of Health and Welfare, Jönköping University, Jönköping, Sweden

**Keywords:** participation, inclusion, physical education, disability, secondary school

## Abstract

**Introduction:**

The objective of this study is to describe how Physical Education (PE) teachers work to facilitate participation for students with disability in compulsory, mainstream inclusive, secondary school. Inclusive school-based Physical Education (PE) is an important context for students to share the benefits of physical activities with peers, especially for students with disability whose opportunities for participation in extracurricular physical activity are limited.

**Methods:**

Two focus group interviews were performed with eight experienced PE teachers who teach students with disability in regular PE. Qualitative content analysis was used to analyze the interviews. Two themes emerged, the importance of having a structured and welcoming environment and the need to adapt the PE environment.

**Results:**

Student mastery experiences is emphasized and achieved by teaching approaches encouraging peer collaboration before physical performance and competition. Key aspects to participation for students with disabilities are inclusive mindsets, proper preparation, and adaptations. Special arrangements when participation in-class is not possible require regular and close teacher-student communication and, when needed, additional support.

**Discussion:**

Experiences of participation are important matters for further advancement of equality and social inclusion for students with disability. Schools need to develop support structures to provide students with disability with “real-life” experiences that optimize participation.

## 1 Introduction

Physical Education (PE) teachers face challenges to create and implement appropriate learning environments for students with disability to participate and feel included in regular school-based PE (1). Teacher attitudes toward inclusion are affected by knowledge about various disabilities, type and degree of disability, class size, teaching assistance, and peer attitudes (2). Inadequate training, lack of training and experience of how to successfully include students with disability are reported in literature as reasons why PE teachers are negative to inclusion (3). For students with disability compulsory school-based PE is an important context to gain the benefits of physical activity (4), especially since opportunities for participation in extracurricular physical activity are restricted (5). Studies show that students with disability may experience participatory gains in inclusive PE, but also that they are less active and feel excluded when participation is restricted (6–9). Inclusive education is stipulated in international policy documents (10–14) to increase participation, community, compassion, and respect for diversity (15). However, discrepancies remain between policy and practice. How diversity is expressed in policy documents vary and therefore provide little guidance to PE teachers on how to implement inclusive practices (16). Differences have been found in what role teachers attribute PE, emphasizing PE as being either an educational or a physical subject (17). Research findings from Scandinavian countries show that PE is dominated by physical activities influenced by competitive sports, which may exclude or negatively impact for example students with disability (18). Additionally, there are challenges concerning insufficient support to properly accommodate students with disability in PE, which limit the effects of policy documents (19).

School-based PE is a context where norms, values, and attitudes can be questioned, which can advance equality and social inclusion for vulnerable groups such as persons with disabilities (20). Inclusive PE practices focus on capability and community support (21), and have a strength-based approach that provides extended opportunities for learning (22). A 15-year follow-up study from Morley et al. (23) about inclusive PE, showed that little had changed in teachers’ perceived ability to include students with disability in regular PE (24). According to Hutzler et al. (25) who conducted a narrative review of PE teachers' attitudes and self-efficacy toward inclusion there are a number of environmental, contextual and individual factors that affect teaching behaviors. It was concluded that teacher attitudes and self-efficacy toward inclusion are interrelated and may work as moderators to inclusive practices, i.e., both facilitators and barriers. Additionally, more research is needed on interventions aiming at changing teachers' attitudes toward inclusion (25). Although showing positive attitudes toward modifying PE activities to fit individuals' special needs, closer collaboration between PE teachers and special educators is needed to inform each other about diversity and inclusive teaching strategies (26).

In the perspective of students with disability mere placement into regular PE settings does not mean inclusive experiences if instructional approaches are unchanged (7). Secondary school students' developmental processes in PE was studied in a 3-year longitudinal study (27), based on social cognitive theory (28). Academic (PE-specific) self-efficacy refers to feelings of competence and capability when successfully completing a task (29). The strongest predictor of future performance is *mastery experiences*, but also sources such as *vicarious experiences* (observing role-models), *verbal persuasion* (encouragement), and *physiological states* such as anxiety and stress contribute to individuals' sense of self-efficacy (30). Compared to students without disability (*n* = 420) individual self-reports showed that students with disability (*n* = 30) perceived lower PE-specific self-efficacy both in year 7 and year 9 (31). Observing the PE context to discern differences in teachers' ability to align curriculum with their teaching practice (high-/low PE teaching skills) all students showed higher engagement in high-level PE teaching conditions. Students with disability were more frequently observed to be in close communicative proximity to their teachers in high-level teaching (32).

PE-specific self-efficacy is closely related to the will to participate in PE (31, 33) and participation can be seen as an expression of inclusion (34). Participation is defined as “involvement in a life situation” (35). To conceptualize participation, there are two key components: being physically present and being actively involved in the activities while being there. Presence can be measured as frequency of attendance. Involvement includes elements of engagement, motivation, persistence, social connection, and level of affect (36). First, one fundamental component in inclusive practices is that disability is celebrated as an asset, not a problem (37). Teachers carry the responsibility to differentiate their instructions to heterogeneous groups of diverse learners. Consequently, the student is relieved from the sole responsibility of adapting his/her learning style to the teaching style offered (38). Mindsets affect the actual classroom practice, and teachers who have limited perceptions of students’ ability or understanding of their specific needs are less accommodating in their teaching approach (39). Secondly, assistance is a crucial component that may facilitate student learning and peer interaction (40). Issues concern assistants' preparedness, qualifications, and training to support students' specific needs properly (41). Individual assistance from non-PE qualified teachers may be inadequate and inappropriate (42). Excessive student-assistant proximity may for example hamper peer interactions (43), which may result in loss of social participation and control for students with disability (44). Thirdly, environmental adaptations may be required to gain access to educational contexts, products, and support services (45).

The voices of students with disability should be honored and a learning environment with student cooperation should be promoted (46). Previous research shows that learning environments that promote mastery experiences, as opposed to competitive environments, improve self-efficacy in PE (31) and motivational outcomes such as self-esteem, affective states, practice strategies and moral attitudes (47). The International Classification of Functioning, Disability and Health (ICF) (35) provides a biopsychosocial framework to understand relationships between disability and activity, and their interaction with environmental factors (48). Based on the ICF, five environmental dimensions were described by Maxwell et al. (49) as a research tool for investigating inclusion. The five A's: acceptability, adaptability/accommodability, affordability, accessibility, and availability can be used to evaluate how to facilitate participation in PE. Acceptability involves the social environment, whether presence is accepted by oneself or others. Adaptations/accommodation may be required to meet the needs of persons with disability. Affordability concerns whether it is worth the time or energy to participate. Accessibility refers to whether the context provides opportunity to participate and, finally, availability to whether the activity is offered or not.

Inclusive mainstream education in Sweden is compulsory to year nine and regulated by the national curriculum (50). The PE syllabus has a health-directed focus with the purpose to provide opportunities for students to develop skills and abilities necessary for lifelong physical activity. Core contents in PE are movement, health and lifestyle, and outdoor life and activities. In a quality report The Swedish Schools Inspectorate (51) summarizes that PE in secondary school is dominated by the core content movement. According to (52), Swedish PE teachers describe themselves as problem-solvers, safety creators, and organizers. Students with disability, compared to students without, were more frequently observed to be near the teachers (32). Either the student sought teacher support, or the teacher stayed near to be able to support, this finding indicates that teacher proximity is a necessary source of special support for the participation of students with disability in regular PE. School-based PE has the potential to provide learning experiences with positive social interactions, enjoyment, and relevant challenges to develop motor skills (53). However, school-based PE also holds environmental challenges to students with disability. Lessons encompass transitions to various sports facilities and activity specific equipment. Although inclusive education has been the norm for Swedish compulsory education for decades, The Swedish Schools Inspectorate (51) still identified individually adapted solutions for students with disability as one area in need of special attention. There is plenty research about teacher attitudes towards inclusion in PE (2, 3, 9), but teaching strategies for how to create positive learning opportunities for students with disability are needed (1). Considering the importance of attitudes and self-efficacy toward inclusion (25) PE teachers are a key environmental factor in facilitating inclusion. Focusing on environmental factors, the aim of this study is therefore to describe how PE teachers work to facilitate participation for students with disability in compulsory, mainstream inclusive, secondary school.

## 2 Materials and methods

### 2.1 Design

This study has a qualitative design where data have been collected in focus group interviews (54) and analyzed using qualitative content analysis (55). The current study is part of a comprehensive research project with 450 students from 26 schools and their PE teachers. Students with diagnosed disabilities in regular schooling were specifically targeted. The three-year longitudinal research project in mainstream inclusive secondary schools explored student self-efficacy and participation in relation to perceived functioning and final PE grades. Student questionnaires were distributed, and data collected year 7 and year 9 (31, 33, 56), and PE lessons were observed year 8 (32). Teachers' self-rated their teaching skills in questionnaires year 7 and inclusive teaching skills were observed year 8 (32).

### 2.2 Participants

All PE teachers in the comprehensive research study (*n* = 22) were contacted and invited to participate in focus group interviews. The sampling of PE teachers was random in the sense that students with disabilities (*n* = 30) integrated into regular schools were recruited first. In a second phase schools (*n* = 26) and PE teachers consented to participate. Four male and four female teachers who worked in the catchment area of a university in southern Sweden agreed to participate physically. Two teachers worked at city schools with more than 550 students, the others worked at schools with less than 300 students in regional towns. Three of the PE teachers had more than 16 years of teaching experience, four 6–15 years, and one less than 5 years. All teachers taught students with diverse types of disabilities in regular PE.

### 2.3 Data collection

Focus group interviews were used to gather diverse perspectives (54) on inclusive teaching strategies practiced in everyday teaching. Two semi-structured focus group interviews with four participants in each were conducted The focus group interviews were conducted without distraction. Before the interview, the participants were given verbal and oral information about the study. After having consented, the participants were invited to talk about how they work to facilitate participation for students with disability in regular PE, i.e., compulsory, mainstream inclusive, secondary school. A semi-structured interview guide based on previous data collection, i.e., student questionnaires (56), teacher questionnaires and observations of PE lessons (32) guided the focus group interviews. The interviews started with a question on how the teachers vary lessons to differentiate their teaching in general. The teachers were then requested to elaborate on more specific questions about how they plan and organize their lessons to meet the needs of students with disability, adaptations, modifications and support. Participants were encouraged to resume the discussion where others stopped and supplement with examples from their own experiences. The interviewer then probed participants to elaborate on more specific teaching realities covering areas of adaptations, support, and special arrangements. An assistant took notes and asked participants to exemplify and give further details to certify that the questions in the interview guide were enriched. The interviewing researcher and the assistant were both experienced PE teachers involved in PE teacher training at the university where the interviews were conducted. The interviews that lasted between 1.5 and 2 h each were audiotaped and transcribed verbatim. Transcriptions were then sent to the participants for confirmation of validity.

### 2.4 Analysis

Qualitative inductive content analysis was used to analyze the data (55). In the first step the authors read the transcripts of the interviews several times to get an overall understanding of the content. In a second step, meaning units, i.e., text that corresponded to the study purpose was identified manually and copied to a coding sheet. Meaning units that were too short were avoided to limit the risk of losing context and meaning. Each meaning unit was then in a third step condensed, which means that text that was repeated or not considered relevant was removed. Then, in the fourth step the condensed meaning units were labelled with a code. The code was close to the original transcript to keep a low level of abstraction. The study aim guided the coding process. Based on content similarities and differences the condensed units and codes were in a fifth step reviewed and grouped into sub-themes. Themes then emerged by combining sub-themes (see Table 1). The analysis went from parts to the whole and vice versa. All steps of the analysis were reviewed and discussed between the authors until consensus was reached.

**Table 1:** Example from the coding sheet.

Meaning unit	Condensed meaning unit	Code	Theme	Sub-theme
Talk to the student beforehand to prepare what will happen in class. The student needs to know what.. that comes, what the lesson contains. I can write on the board, but it doesn't help and then a little closer contact during the lesson, without making it obvious to the others. Classmates often know who has difficulties, but one can always try to close the gap…so that it doesn't show	Talk to student beforehand to prepare. Student needs to know what the lesson contains. I can write on the board, but it doesn't help. Closer contact during the lesson, without making it obvious to the others	Adapted support	To ensure that all students know what to expect	The importance of having a structured and welcoming environment
I can't let a student with visual impairment go alone in the woods because then she will stumble on roots. She has been with me and set up checkpoints, for example, or is with me and draws courses to check how much she has understood of orienteering. A student with autism, I have to think about who I pair her with, certain activities are more difficult. She doesn't like balls and during ball drills I want everyone to have a ball each, but then she disappears from the lesson, so I have to try to make sure that she stands in a corner and I kind of protect a little, like this (shows)	Can't let a student with visual impairment go alone in the woods because then she will stumble on roots. She has been with me and set up checkpoints or draws courses to check how much she has understood of orienteering. A student with autism, I have to think about who I pair with. Certain activities are more difficult. She doesn't like balls and during ball drills I want everyone to have a ball each, but then she disappears from the lesson, so I make sure that she stands in a corner. I kind of protect a little	Adapted lesson content	To modify the activities and provide special support	The need to adapt the PE environment

### 2.5 Ethical considerations

Ethical approval was obtained from the Ethical Review Board, Linköping, Sweden (2013/508-31). Students in the comprehensive study were never identified by name in the interviews, instead the teachers referred to types of disability and included examples from previous experience.

## 3 Results

The analytical process resulted in two themes and five sub-themes, see Table 2.

**Table 2:** Identified themes and sub-themes.

Theme	Sub-theme
The importance of having a structured and welcoming environment	To create an inclusive classroom climate
	To keep comprehensible communication
	To ensure that all students know what to expect
The need to adapt the PE environment	To adjust teaching style and lesson content
	To modify the activities and provide special support

### 3.1 The importance of having a structured and welcoming environment

A structured and welcoming environment is presented in the three sub-themes *to create an inclusive classroom climate, to keep comprehensible communication* and *to ensure that all students know what to expect*.

#### 3.1.1 To create an inclusive classroom climate

For students with disability to participate in the PE lessons, a welcoming classroom climate was important. Most importantly, according to the teachers, was to create a climate with collaboration rather than competition. The PE teachers emphasized the importance of involving peers to assist students with disability. The teachers tried to arrange the lessons so that students with disability could always perform the exercises near their friends. The teachers stressed that when focusing on assisting a peer with difficulties, more energy was spent on learning and less on watching other classmates' accomplishments.

…collaborating with a friend who you trust, you listen to the friend, maybe even more than to the teacher…

A welcoming classroom was enabled by using different strategies. One strategy was to provide balls while waiting for the lesson to start. The first students entering the gym could choose to play around with the balls and others could choose to socialize with each other. This was also valuable student-teacher time when information about prerequisites for today's participation was shared, diverse needs were discussed, and terms for participation were agreed.

The students get a positive feeling, they never come late, they are always there and then when the lesson is about to start, the balls are put away. Some students get rid of energy, and I can spend time to chat with other students…

Another strategy was to kick-start the lesson by giving brief instructions that activated most students. This would give the PE teacher time to spend on additional instructions that made it possible for students with disability to join in as quickly as possible. Often, further instructions needed to be shorter and stricter, like “go there! do this! do that!.” Sometimes the PE teachers demonstrated how to do the exercise practically or how to use different equipment. In doing so, the students could imitate exercises and use the equipment properly. It was highlighted that it was difficult to give instructions once the student with disability was moving without stigmatizing the student owing to limited ability. One solution to this problem was for the teacher to “shadow” the student, i.e., to try and keep communicative proximity to these students.

And then some closer contact, without making it obvious to the others. Classmates often know who has difficulties, but one can always try to close the gap…so that it doesn't show.

#### 3.1.2 To keep comprehensible communication

The PE teachers described that many students in need of special support needed more detailed information to make them want to participate and feel engaged in the PE lesson. Generally, the PE teachers made both long-term plans for the entire semester but also detailed weekly plans to prepare the students for upcoming activities. This information was spread differently depending on what communication tools the school used: it could be on paper, in class, in home classrooms, at meetings where parents were invited or via electronic platforms. Communication via e-mail to parents of students with disability was sometimes needed to ensure that these students were adequately prepared for upcoming events. Different strategies were used to make the information comprehensible in class. One strategy was to gather all students before the lesson. To facilitate participation for students with disability, one teacher used white-boards and/or projectors to visualize oral and/or written instructions. She experienced that she reached more students, irrespective of disability or not, and started to use visual supports more frequently.

I had a whole class preparing year 8 for an outdoor event, where I drew a sort of map on the whiteboard of what was going to happen. I drew pictures of equipment and everything which is usually fun because I can't draw. Students laugh, then it sticks, and they remember. Everyone brought a rubbish bag! Imagine that!

Gatherings were used to explain today's activities and to legitimize the lesson content. The PE teachers tried to explain the purpose by referring to the syllabus or general health aspects, for example, that the students would improve their well-being, fitness, strength, and motor skills by taking part in the PE lesson. Another strategy to motivate the students to participate in PE was to give authentic examples that emphasized the importance of the skills to be practiced.

…like yesterday, they were going to perform a group play about a fictitious accident presenting a feasible solution, I came late to class since I had been to a fire emergency and then of course they were curious, which makes it easier. I can give plenty of examples from reality and then they realize. Everyone needs to know this, not only talk, and read this paper and you'll understand…

#### 3.1.3 To ensure that all students know what to expect

When ending the class, the PE teachers expressed that it was important to prepare for the next lesson. Generally, the PE teachers therefore gathered the students to evaluate today's lesson and to update them about upcoming lessons. Several reminders were needed to prepare for extraordinary events, i.e., future outdoor field days or events that require specific facilities or equipment, for example, swimming, orienteering, and ice-skating. After the finishing gathering, face-to-face information with students with disability was sometimes needed to make them feel prepared, safe, and secure for the next PE lesson. The PE teachers experienced that new activities might cause unnecessary stress, especially for students with neuro-developmental and/or intellectual disabilities. Examples of how they prepared these students before introducing a new activity were to explain rules, to practically demonstrate the activity and to provide opportunities to test specific skills or drills.

All students have a need to know in advance what will happen but students with disability want to know “a little deeper”. As a teacher I need to tackle this for these students to feel safe from the start.

At one school the students were provided with their own school computer and an electronic platform was used as the main source of information. This offered opportunities for the teachers to post films and links. The students could then practice for example gymnastics or dance choreographies in advance or after the class whenever they wanted.

I always write, so that they know what is going to happen in the next two weeks and then in class it's oral instructions and practical demonstrations. After that they can use their computers, like now for example when we do gymnastics, I have posted films with 50 different exercises that they can watch many times

### 3.2 The need to adapt the PE environment

Adaptations that are needed in PE are presented in the subthemes, *to adjust teaching style and lesson content*, and *to modify the activities and provide special support*.

#### 3.2.1 To adjust teaching style and lesson content

Firstly, the PE teachers tried to adjust their teaching style. One way was to organize two parallel activities, one in a small group format and the other as a self-sustaining whole group activity. Meanwhile, monitoring the latter, they could focus on student progression in the smaller teacher-led group by individualizing instructions, support, feedback, and feedforward. To avoid stigmatization, they intentionally split students into small groups or pairs so that students with disability were always accompanied by at least one friend. Secondly, teachers made individual adjustments of lesson content to facilitate participation in PE for students with disability, preferably within regular PE class, but occasionally in a separate setting. The lessons were sometimes adjusted for students with disability by shortening the time span for the activities. For example, instead of playing the same game the whole lesson, one game could be played for 10 min, then be modified, or changed to another game and played for another 10 min and so on. By doing so, full participation was facilitated in at least some of the activities. Another strategy to adapt the teaching was to use a part-part-whole teaching style. This strategy has been proven to be successful when working with students with intellectual disability in adapted PE at special schools. Before playing softball for example, the students practiced striking the ball, catching the ball and how and when to run. When all parts had been practiced, the parts were put together into a game of softball. However, not all PE teachers planned their adjustment strategies in advance. Some PE teachers planned the same activity for all students without special arrangements. Once faced with a situation where participation was not possible, some teachers solved problems as they emerged and provided alternative solutions in relation to students' challenges and type of lesson. For example, when energy levels became too low, students with disability could take a rest or the teachers would provide individual motor skill activities in relation to lesson content.

I might use balance exercises, it works because both have poor motor skills, and the student with heart failure is also overweight, so then partly to train some motor control, with her, do some simple muscle-strengthening activities, which in short intervals, she manages to conduct

The teachers who solved participation problems as they emerged also said that they were more willing to adapt their teaching in advance if the student showed interest in PE and suggested alternative solutions themselves.

#### 3.2.2 To modify the activities and provide special support

The PE teachers had a genuine willingness to modify activities during PE lessons to facilitate participation for students with disability. They strived to differentiate every PE lesson to optimize participation for all students. Examples of such general modifications were to provide clear rules, challenges at altered levels of difficulty in circuit training, alternative routes in obstacle runs and a variety of exercises and drills in gymnastics. Another strategy to differentiate teaching was to introduce Paralympic sports e.g., goalball that all students can play blind folded. Although the PE teachers had inclusive intentions in regular PE classes, this was not always possible. Activities were sometimes modified to facilitate individuals' participation, with or without personal assistance. Communication with the student was then vital to map possibilities and find feasible solutions that could be performed either integrated into the class or separate from the class.

I read in her blog that she was in pain after regular PE, so I talked to her and drew up a plan. Now she swims one lesson (with personal assistant) and I structure the other lesson so that she can participate to the best of her ability half the lesson, and the other half she does a workout program that she has constructed herself. (Girl, 14 years old, electric wheelchair user)

The PE teachers experienced that schedule-breaking activities with new group constellations, new environments, unexpected new situations, and unfamiliar teachers could be stressful to any student, but particularly for students with neuro-developmental disorders (NDD). According to the PE teachers, outdoor field days with winter/summer activities required innovative modifications. The PE teachers experienced that it was crucial to give additional information to students, parents, and staff about clothing, special equipment, and transportation back and forth for example. A variety of options were offered, including at least one activity in which anyone could participate in e.g., hiking, swimming, and sledding. Despite teachers' extra work-load and stressful new situations for some students, the teachers praised outdoor field days since they provided opportunities to fraternize on equal terms across classes. The most important aspect to enable participation for students with disability was the provision of additional assistance. In some cases, parents were engaged to assist their child during the outdoor field days, e.g., alpine skiing. In others, for example, a school staff janitor was responsible for one student or a small group of students to facilitate participation.

If it's winter, some students ski, some skate, and everything is nearby, then sometimes we gather for a BBQ so that they (students with NDD) at least can attend having lunch…that's one thing one can do…rather than staying home alone.

## 4 Discussion

Inclusion has been the norm in Swedish mainstream schooling for some time now and the immediate response from the PE teachers on how they facilitate participation for students with disability was *I don't really know; I just do it!* However, findings from this study show that participation in PE for students with disability is facilitated by environmental adaptations that concern classroom climate, communication, student preparation, adjusting teaching styles and lessons, as well as modification of activities and special support.

### 4.1 Inclusive PE practice

Participation of students with disability in PE may be facilitated or hindered owing to personal factors of restricted functioning or to factors in the environment. Environmental aspects of how inclusive PE practices were enacted will be discussed with the five A's: acceptability, adaptability/accommodability, affordability, accessibility, and availability (49).

#### 4.1.1 Acceptability—fostering tolerance and respect

Acceptability relates to the social environment and refers to whether the presence of students with disability is accepted by themselves or others (49). In our findings the PE teachers pronounced an inclusive mindset. They had a genuine willingness to facilitate participation for students with disability and tried to create a classroom climate that recognized and accepted diversity. According to Falkmer (57) teachers' attitudes towards students with disability and good teacher-student relations promote positive peer interaction.

Primarily, collaboration between peers was emphasized by the teachers in this study. By collaborating with a peer, the teachers provided students with disability an opportunity to participate in PE that gave them *vicarious experiences* (observing role-models). Peers could encourage their efforts, reduce their anxiety so that they dared to make an effort, practice and persist practicing to successfully complete a task. Successful experiences can improve a general sense of capability (58), which may spread across other areas of competency and affect the overall functioning of students with disability (28). Additionally previous research shows that assisting peers with disability promotes levels of physical activity both for students with and those without disability (59).

However, contradictive findings show that PE is dominated by the core content movement (51), which often include activities influenced by competitive sports (18). A prerequisite for experiencing participation is that the student is physically present (36) but persons with functional restrictions can seldom (or never) participate on equal terms in competitive climates where physical performance is rewarded. In addition to being physically present, active involvement while attending the PE lesson is crucial to experiencing participation (36). Our findings showed teaching practices of acceptability, where efforts were made for students to feel welcome, and time was allocated for communication about participatory concerns. Additionally, how acceptability was promoted by preparing students with disability for the lesson, offering challenges at altered levels of difficulty, and how facilitating participation made out the ground for how the lesson content was planned, organized, and adjusted.

To differentiate instructions successfully there needs to be a coherence between teacher mindsets and actual classroom practice (39). The teachers in this study strived to create positive learning environments with tolerance and respect for diversity. Findings that are in line with international policy documents about inclusive education [e.g., (12)] and curricular intentions of providing opportunities to learn for lifelong physical activity (50). Observations of PE lessons (32) in the comprehensive study demonstrated that students with disability were equally highly engaged as their peers. Highly engaged students indicate that the teachers have adopted an inclusive teaching style and created a learning environment of acceptability, where effort is encouraged before competition. According to previous research, learning environments that promote mastery experiences boost self-efficacy in PE (31), self-esteem, affective states, practice strategies and moral attitudes (47).

#### 4.1.2 Accomodability/adaptability—creating meaningful learning experiences

Adaptability refers to whether the activity is adequately adapted to accommodate students with disability (49). Firstly, the teachers strived to adapt their teaching strategies to facilitate participation integrated in class. A general approach to differentiate teaching was to provide a variety of exercises at diverse levels of difficulty and alternative activities or routes in e.g., obstacle runs. More specific strategies were to introduce Paralympic sports, shorten the time span and change traditional team sports into more playful games. A successful strategy to provide individualized feed-back and support was to organize the lesson into one teacher-led small group activity, and one self-sustaining and easily monitored whole group activity. The small groups were intentionally composed by the teachers to ensure that students with disability could practice skills with friends they trusted. Visual supports were established into regular PE since also other students without disability proved to benefit from an additional source of information.

Secondly, when functional limitations hindered participation, the lesson content had to be modified. The teachers expressed a wish for further advice but experienced a lack of competence in special educators concerning modifications in a practical subject like PE. Similar findings in Finland show that closer collaboration between PE teachers and special educators is required to optimize learning opportunities for students with disability (26). Frequent check-ups and regular communication with students with disability were highlighted by the teachers in this study. Some teachers made plans in advance with the student with disability while others dealt with problems as they emerged during class. According to Bredahl (60), participation is enhanced when adaptations are made in mutual communication where students with disability are offered a choice of alternative activities, either integrated in or separate from the class. Haegele and Sutherland (61) report that students with disability experience meaningful learning when accommodations are made during class.

#### 4.1.3 Affordability—making participation worth-while

Affordability concerns a person's willingness to invest effort, or energy into participating in an activity (49). Challenging environments and functional limitations sometimes required additional assistance. Our results showed one example when an electric wheelchair user who due to pain after regular PE-class needed additional adaptations. Rather than to adhere to the image of disability, an inclusive PE practice recognizes students' strengths (22). The teacher honored the student's voice (46), recognized strengths and abilities and together they planned affordable participation. In this case support from a personal assistant was required, or participation would not have been possible at all. In-class participation was preferred but if not possible, the student could choose from activities separate from the class. Lesson content was modified in collaboration with the assistant, which optimized opportunities for participation and made PE participation affordable, i.e., worth both the effort and energy for student engagement.

Although Haegele (7) questions the inclusiveness of regular PE a change towards more inclusive PE practices seem to be taking place. In the most recent articles about student experiences of inclusion in PE Rekaa et al. (9) found students with disability who “love PE”. Swedish PE teachers describe themselves as problem-solvers, safety creators and organizers (52), which was supported in this study. Findings in this study show that the teachers' have changed attitudes and self-efficacy toward inclusion (25) and implemented inclusive teaching strategies with a primary aim to facilitate in-class participation. The teachers gave plenty of examples of innovative modifications to facilitate participation for students with disability, including additional assistance from parents and janitors.

Outdoor field days are commonly organized by PE-teachers within the subject's core content, outdoor life and activities (50) and entail activities for students, colleagues, and co-workers. Notably, outdoor field days concern the whole school, but PE-teachers experienced additional work-load due to insufficient school support when facilitating participation for students to whom outdoor field days were especially stressful. Previous research shows that collaborative strategies improve sense of belonging, school satisfaction, and self-efficacy for new experiences among students with disability (62). Although the teachers claimed that students' participatory gains made their extra work-load worthwhile, they wished for more school support when planning adjustments to facilitate affordable participation.

#### 4.1.4 Accessibility—setting up safe PE environments

Accessibility concerns whether access to the facility or to the specific context is possible (49). The teachers in this study did struggle to make their lessons accessible and facilitate participation for students with disability in regular PE. This finding is similar with previous findings that teachers are positive to inclusion but find it impossible to achieve (9). The strategy to try and stay near to be able to assist whenever support was needed confirms previous observations where PE teachers seemed to be the primary source of support for students with disability in regular PE (32). By keeping close communicative proximity to students with disability teachers can encourage them to achieve *mastery experiences*. Prior accomplishment is the strongest predictor of future performance (30). Staying near, teachers can also promote the other sources of self-efficacy by acting as role-models, encouraging effort and performance, and reducing anxiety.

Preparation and communication were vital to make PE activities accessible to students with disability, especially when new unfamiliar activities were introduced. PE instructions commonly require close teacher-student proximity and may require visual support, repeated oral and kinesthetic information in various forums, and additional face-to-face information to make students with disability feel safe. Accessibility also increased when the teachers created short “timeslots” where students with disability were given additional instructions. These timeslots took place in before-class activities, by kick-starting lessons to activate typically developing peers, by staying near students in need of special support during class or by explaining and demonstrating future PE activities after ending a lesson.

Swedish gym facilities are generally supplied with elevators, hearing loops, technical aides etcetera to be accessible to all, but there are situations or activities where participation is impossible. Accessibility to outdoor environments e.g., forest or gravel-yard may be problematic for students with physical disability to be able to participate. Accessibility to school-based PE may also be hampered by prevailing norms and values of a fit body (9, 63) and of traditional competitive sports (8). The common teaching approaches with peer collaboration may be helpful to gain access to PE-specific contexts, but assistance from non-PE qualified friends may be inadequate and inappropriate (42). Events like swimming, orienteering, and ice-skating often take place outside school premises and accessibility may require personal assistance or students with disability will lose social participation and control (44).

#### 4.1.5 Availability—participation promoting support structures

Availability relates to the physical environment and refers to whether the activity is offered or not (49). The main issue concerning availability in this study was the lack of sufficient support. Previous studies show that assistants are rare in PE (3, 32) and insufficient support may make PE lessons unavailable and thereby limit the effects of policy documents (19). In the current study there was only one teacher who experienced sufficient teacher support and student assistance. Availability means prerequisites for teaching practice that PE teachers are not in control of but need to be taken into account when teaching PE. In the absence of support, the PE-teachers developed innovative solutions to make regular PE available to students with disability. To facilitate participation and effectively include students with diverse needs they implemented clear classroom structures, new teaching strategies, and adaptations (both general and specific).

Participation-based interventions for youth with disability emphasize the merit of meaningful “real-life” experiences. Results from previous studies show promising synergy effects of community participation on physical, cognitive and affective body functions (64) and on goal attainment, social self-efficacy, and engagement (65). Resource allocation in school is a matter of concern. Due to lack of adequately trained support team and allocation of assistance favoring academic performance before participation in PE, the PE teachers do what they can to facilitate participation for students with disability. School support to make everyday situations like school-based PE available to students with disability may need to target participation to be effective. Being able to attend and experiencing engagement while attending (36) in school-based PE may underpin further advancement of equality and social inclusion (20). Collaborative teaching approaches (62) are required to optimize participation in PE for students with disability.

## 5 Strengths and limitations

Findings from this study can be used in pre-service teacher training and complementary in-service training to develop inclusive PE practices and teaching strategies. The participants were taking part in a comprehensive study focusing on students with disabilities. It is reasonable to assume that the participants had considered inclusion and therefore paid extra attention to how they facilitate participation in PE for students with disability. Teaching conditions were similar, they could easily relate to each other and add experiences from their own practices.

There are limitations in this study that need to be addressed. There were only two focus interviews but the participants in this study had plenty of teaching experience. Focus interviews provide the potential for getting further into a subject owing to the interaction. Individual interviews with teachers, and students' views of their everyday life situations in PE may have enhanced the overall picture. Moreover, only eight of the invited twenty-two teachers agreed to participate in the focus interviews, possibly those most favorable of inclusion.

## 6 Conclusion

Environmental barriers cause exclusion by limiting or preventing a person with disability from fully participating in school-based Physical Education (PE). Restricted participation occurs when disability is treated as a problem, and when adaptations and support are insufficient. The five A's, acceptability, affordability, adaptability/accommodability, accessibility and availability provide PE-teachers with a useful tool to assess their PE environment and evaluate how to provide equal access to their practice. A key environmental factor to facilitate participation for students with disability in regular PE is the teacher. PE teachers' views in this study show that a classroom climate with respect and tolerance toward diversity is vital. Additional instructions are often required for students with disability to feel prepared and safe in regular PE. Collaboration with friends they trust reduces anxiety and promotes experiences of mastery. Close teacher-student communicative proximity provides opportunities for encouragement and additional support. In-class participation requires adapted teaching strategies, modified activities and extended instructions, e.g., visual support. Concerns are raised regarding school support to optimize participation in PE. Schools need to develop support structures that focus “real-life” participation experiences to facilitate positive outcomes of regular PE for students with disability. Effective school support structures with adequately trained support team can supply PE teachers with collaborative teaching approaches and practical guidance on how to optimize learning opportunities in PE for students with disability. Further practice-based research is needed on how to adequately support students with disability in PE. Additionally, effects of “real-life” participation experiences on overall everyday functioning of students with disability need further investigation.

## Data Availability

The raw data supporting the conclusions of this article will be made available by the authors on request, without undue reservation.
